# Risk Assessment of Nasal Cavity Perforation in the Maxilla: A Virtual Implant Placement Study Using Cone Beam Computed Tomography

**DOI:** 10.3390/diagnostics14141479

**Published:** 2024-07-10

**Authors:** Doğan Ilgaz Kaya, Samed Şatır, Beyza Öztaş, Hasan Yıldırım, Ahmet Aktı

**Affiliations:** 1 Department of Oral and Maxillofacial Surgery, Faculty of Dentistry, Karamanoglu Mehmetbey University, 70200 Karaman, Turkey; 2 Department of Oral and Maxillofacial Radiology, Faculty of Dentistry, Karamanoglu Mehmetbey University, 70200 Karaman, Turkey; samedsatir@kmu.edu.tr; 3 Department of Oral and Maxillofacial Surgery, Faculty of Dentistry, İstanbul Kent University, 34433 Istanbul, Turkey; beyzadonmez@kmu.edu.tr; 4 Department of Mathematics, Kamil Ozdag Faculty of Science, Karamanoglu Mehmetbey University, 72100 Karaman, Turkey; hasanyildirim@kmu.edu.tr; 5 Department of Oral and Maxillofacial Surgery, Faculty of Dentistry, Selcuk University, 42250 Konya, Turkey; dt.ahmetakti@gmail.com

**Keywords:** cone beam computed tomography, nasal cavity, perforations, virtual implant

## Abstract

Implant surgery in individuals with atrophic maxilla presents challenges, particularly concerning nasal cavity complications such as perforations, implant migration, and airway obstruction. While panoramic radiographs offer diagnostic convenience, their two-dimensional nature limits the evaluation of anatomical structures. Cone beam computed tomography (CBCT) provides a three-dimensional assessment, enhancing surgical planning accuracy and potentially reducing complications. With the aim of understanding the measurement differences between panoramic radiographs and CBCT images, this retrospective study examined CBCT images of patients with severely atrophic maxilla taken between September 2021 and December 2023 at the Ahmet Keleşoğlu Faculty of Dentistry. Virtual implants were placed in various tooth regions using OnDemand3D software. The incidence of nasal cavity perforations and vertical bone height differences between panoramic radiographs and CBCT images were evaluated. For this purpose, vertical bone length measurements in panoramic and CBCT images were compared for the virtual implant placement areas. Statistical analyses, including *t*-tests and ANOVA, were performed to determine significant differences among quantitative measurements, and a chi square test with Bonferroni corrected z-tests were used for possible associations between ratios. CBCT data from 59 patients, totaling 1888 virtual implants, revealed significant differences in implant depths among tooth regions (F = 9.880, *p* < 0.001). Canine regions showed higher perforation risks, especially with 12 mm and 14 mm implants. Panoramic radiographs often overestimated vertical bone height in canine and first premolar regions compared to CBCT measurements, which could lead to increased perforation risks. Radiographic evaluations using CBCT prior to implant surgery in atrophic maxilla cases are crucial to prevent nasal cavity complications. Panoramic radiographs may inadequately represent three-dimensional anatomy, underscoring the importance of CBCT for accurate implant placement and surgical planning. Further studies should consider varying implant sizes and brands to generalize findings.

## 1. Introduction

Implant surgery may cause some difficulties in individuals with atrophic maxilla. Among these, complications related to the nasal cavity can cause situations that are difficult to handle. Some of these have been reported as nasal cavity and nasal mucosa perforations, migration of the dental implant into the nasal cavity, nasal airway obstruction, and rhinosinusitis [1,2,3,4,5]. To avoid complications such as these, it is necessary to have a good understanding of the patient’s maxillofacial region anatomy. Various radiographic methods can be used for this purpose.

Panoramic radiographs provide convenience to dentists for diagnosis and planning with a single image [6]. However, the two-dimensional nature of the images obtained from panoramic graphics limits our understanding of some anatomical structures [7]. Disadvantages of panoramic radiographs include the superposition of important anatomical structures and the inability to evaluate the alveolar crest buccopalatinally. Cone beam computed tomography (CBCT) allows for a three-dimensional evaluation of important anatomical structures of the maxillofacial region. Its superiority has been proven when compared to panoramic radiographs [8]. Understanding the differences between panoramic radiographs and CBCT images is important to prevent complications. Additionally, with some software, it is possible to simulate surgery by placing implants in a virtual environment on CBCT images [9]. This can help predict complications that may occur during or after implant surgery.

There are studies in the literature that evaluate the relationships of virtual implants placed in the software with important anatomical structures such as the mandibular canal, pterygoid space, and nasopalatinal canal using CBCT data [10,11,12]. In these studies, virtual implants of different sizes were placed in the alveolar crest, and their relationships to important anatomical structures were examined. However, no study evaluating the incidence of nasal cavity perforations was found. In their study, Park et al. found that 13 of the 65 patients (26 of 106 implants) evaluated had an unintentional perforation of the nasal cavity. The mean age of the patients with perforation was 50.88; 10 of them were male, and 3 were female [13]. The aim of this study was to evaluate the incidence of nasal cavity perforation by placing virtual implants on the CBCT images of patients with atrophic maxilla. This study will provide insight into the prevention of nasal-cavity-related complications in individuals with atrophic maxilla. In addition, we aimed to determine the possible factors that may cause nasal cavity perforation by comparing panoramic images and CBCT images.

## 2. Materials and Methods

### 2.1. Patient Selection

**Figure 1:**
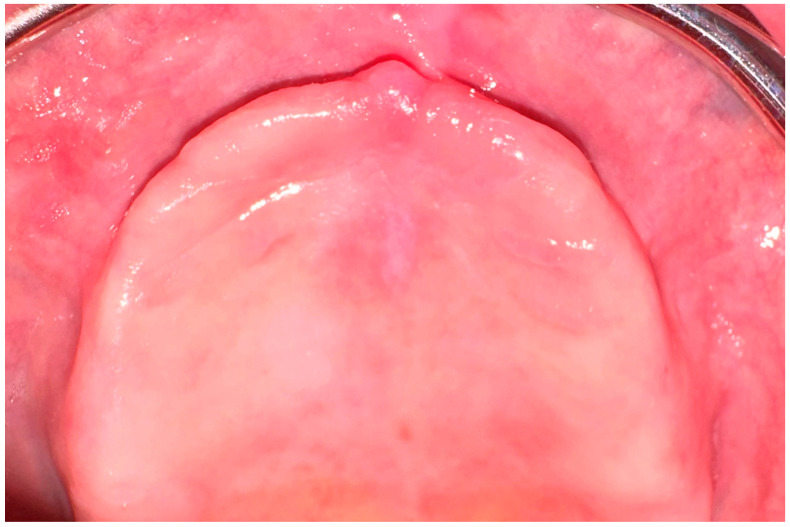
The incisive papilla appears to be localized in the vestibule of the alveolar crest.

This retrospective study was conducted at the Ahmet Keleşoğlu Faculty of Dentistry with the permission of the Karamanoğlu Mehmetbey University Faculty of Medicine Ethics Committee (#06-2023/05). This study was conducted in accordance with the 1975 Helsinki Declaration, as revised in 2013. The images of patients with severely atrophic maxilla who applied to the Oral and Maxillofacial Surgery Clinic between September 2021 and December 2023 and who had CBCT images taken for implant planning were evaluated. Severely atrophic maxilla was determined by the presence of the incisive papilla on the vestibule side of the alveolar crest (Figure 1). All CBCT scans were performed by the same radiology technician using a Kavo OP3d Pro (PaloDEx Group Oy, Tuusula, Finland) device according to the manufacturer’s instructions. The parameters used to obtain the images used in this study were 90 kV, 8 mA exposure setting, exposure time of 17.5–26.9 s, 13 × 15 cm field of view (FOV), and voxel size of 0.320 mm. Patients with atrophic maxilla, with complete edentulism, with a sufficiently horizontal alveolar crest, who had their last tooth extraction at least 1 year prior, and with available CBCT data were included in this study. Patients with pathology in the maxillofacial region or those who underwent surgery for this reason, patients with alveolar crest defects, patients with impacted teeth in the upper jaw, patients with horizontal insufficiency in the alveolar crest, and patients with artifacts in their CBCT data were not included in this study. These criteria were determined by an oral radiologist and a maxillofacial surgeon (S.Ş., D.I.K.).

### 2.2. Radiographic Measurements

#### 2.2.1. Virtual Implant Placement

Virtual implant placement procedures were performed independently by two maxillofacial surgeons (D.I.K., B.Ö.). When there was a disagreement in the measurements made after the implants were placed, a consensus was reached by obtaining the opinion of a third maxillofacial surgeon (A.A.). Due to disagreements in the measurements, the opinion of the third researcher was sought 12 times. The patients’ CBCT data were transferred to the implant planning program (OnDemand3D software(version 1.0.7462), Cypermed Inc., Seoul, Republic of Korea), and implants were placed in the central, lateral, canine, and first premolar tooth regions on the right and left sides. Implants of sizes 3.3 × 8, 3.3 × 10, 3.3 × 12, and 3.3 × 14 (Straumann BLT, Basel, Switzerland) were placed in the specified areas.

Virtual implants were placed in the central, lateral, canine, and first premolar tooth regions parallel to the midfacial midline, taking into account the buccal and palatal alveolar borders. While placing the implants, the nasal cavity was not taken into consideration in the apico-coronal direction. The implant was inserted deeply until its coronal borders were completely within the bone. The implants were angled to match the form of the alveolar crest in the sagittal section.

#### 2.2.2. Parameter Measurement

**Figure 2:**
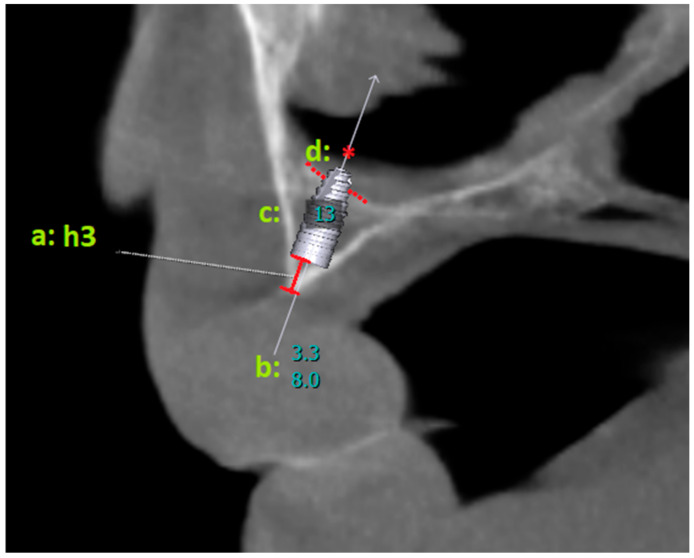
a: h3 shows the distance between the coronal border of the implant and the alveolar crest ridge; b: length–diameter information of the placed virtual implant; c: number of the tooth region; d: * the nasal cavity is observed to be perforated.

For all implants placed, whether the apical parts of the implants perforated the nasal cavity was recorded. The distance between the coronal level of the implants and the alveolar crest ridge (h3) was measured (Figure 2).

**Figure 3:**
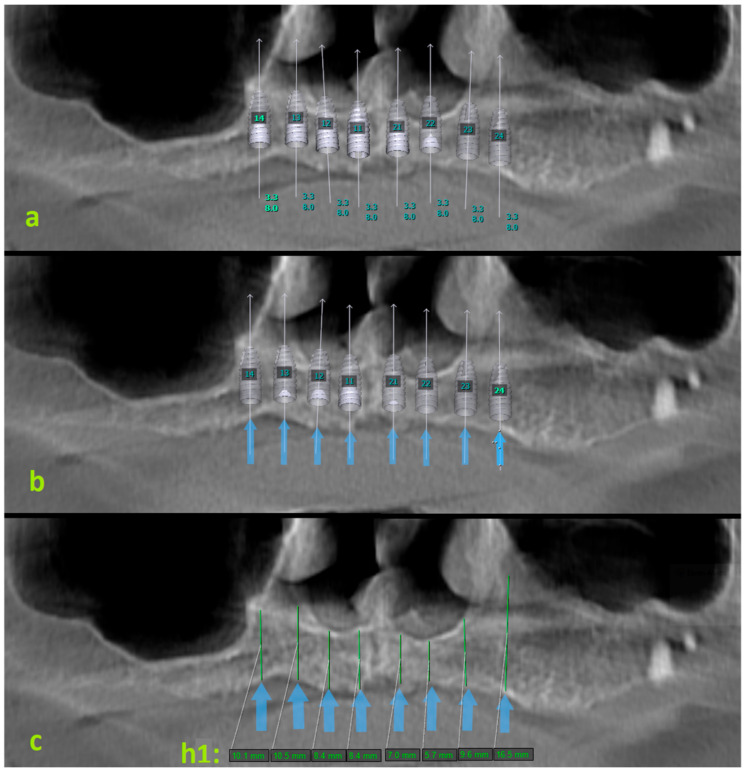
Panoramic radiographs obtained from CBCT images are shown. (**a**): panoramic radiograph of virtual implants placed in the central, lateral, canine, and premolar regions of the maxilla; (**b**): blue arrows mark the virtual implant sites; (**c**): when the virtual implants were removed, vertical height measurements were made on panoramic radiographs from the central, lateral, canine, and first premolar tooth regions (h1).

**Figure 4:**
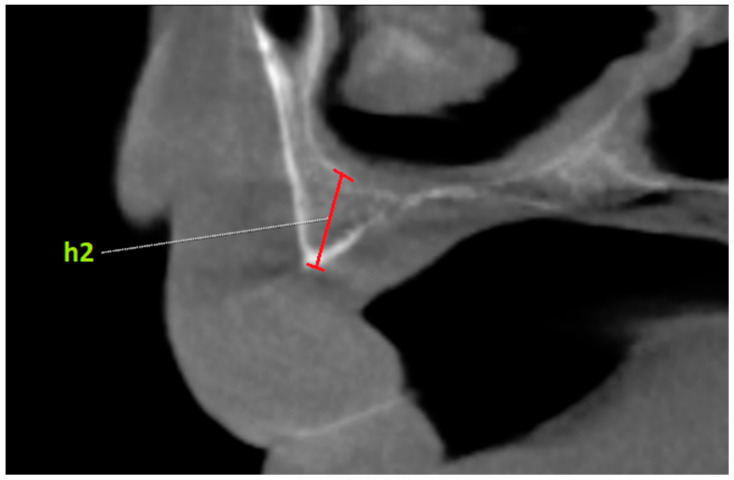
Measurement of the area where the virtual implant was placed in the para-axial section obtained from CBCT. h2: vertical height of the area between the lower border of the nasal cavity and the alveolar crest ridge.

Additionally, the vertical bone height was measured from panoramic radiographs for the areas where virtual implants would be placed (Figure 3). For this purpose, firstly, the areas where virtual implants were applied were marked on the panoramic radiograph. Then, the virtual implants were removed, and the maximum vertical length of the alveolar crest in the marked areas was measured. This value was recorded as h1. Afterwards, the paraxial sections of the marked areas were examined on CBCT. The maximum vertical length of the alveolar crest in the same area was measured on CBCT. This value was recorded as h2. These measurements were compared with the vertical bone height in paraxial sections obtained from CBCT (Figure 4). The purpose of this was to compare the differences between panoramic radiographs and CBCT images (h1–h2).

### 2.3. Statistical Analysis

Initially, the demographic variables were examined, with numerical variables summarized via mean values and standard deviations, while qualitative variables were presented with frequencies and percentages. To compare dental measurements, independent samples *t*-tests were employed to analyze gender differences, and repeated measures analysis of variance (ANOVA) was used for comparisons across different dental regions. The assumption of normality for these parametric tests was assessed using the Kolmogorov–Smirnov test and skewness values, while homogeneity of variances was evaluated using Levene’s test. Multiple comparisons were conducted using the Bonferroni test. Furthermore, the chi square test was utilized to examine the proportional relationships between implant size, tooth area, and perforation status. For fixed implant sizes, the proportional comparisons between tooth area and perforation status were analyzed using the Bonferroni corrected binomial test. The chi square test of independence was applied to examine the frequency distributions of h1–h2 differences with different tooth regions. In addition, a z ratio test with Bonferroni correction was performed to explore possible sub-cell differences between columns. In the power analysis of this study, the effect size was calculated as 0.785 based on the preliminary scores (e.g., h1 value) collected from 12 men and 15 women, indicating that the sample size required for a minimum power of 80% was 27 individuals in each group. Therefore, this study is considered to have an adequate sample. All analyses were conducted using R (version 4.3.2) and MedCalc (version 20), with a significance level set at 0.05.

## 3. Results

In this study, CBCT data of 59 patients were examined, and a total of 1888 virtual implants were placed in eight different tooth regions. According to the results presented in Table 1, the mean age of the 59 study participants was 60.81 ± 11.39 years, and the gender distribution was balanced.

**Figure 5:**
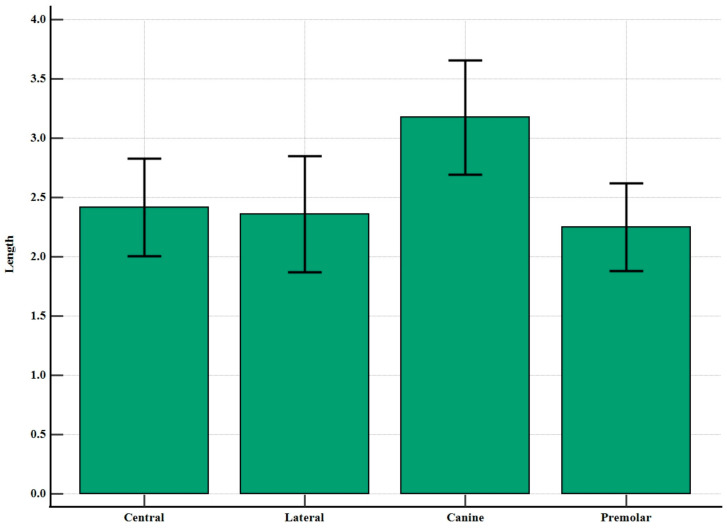
Distribution of h3 lengths at different tooth regions with 95% confidence intervals.

According to the comparison results based on h3 length presented in Table 2, a statistically significant difference was found between the regions (F = 9.880, *p* < 0.001). Multiple comparisons revealed that the canine region significantly differed from the other three regions, while no significant difference was observed among the mean values of the remaining three regions. A visual representation of this difference is provided in Figure 5.

The results given in Table 3 show that the proportional distributions of perforation status and tooth regions are significantly different, and these measurements are not independent of each other (*p* < 0.001). We can state that 12 mm (*p* = 0.045) and 14 mm (*p* = 0.003) implant sizes contributed the most substantial relative effect to this finding, as they constituted the main implant sizes causing the statistical difference. When the implant size was fixed, the rates of occurrence and non-occurrence of perforation in the first premolar region were found to be significantly different, while the rates were similar in the other regions at 10 mm and 12 mm lengths. The rate of occurrence and non-occurrence of perforation was also different in the other regions except for the canine region at a 14 mm length.

Accordingly, 8 mm long implants cause statistically significant less perforation in all regions. The 10 mm long implants cause less perforation only in the first premolar region, while there is no statistically significant difference in other regions. When 12 mm long implants are used, the incidence of perforation is significantly higher for central, lateral, and canine teeth outside the first premolar region. In 14 mm long implants, the incidence of perforation is statistically significantly higher in central, lateral, and first premolar teeth outside the canine (Table 3).

The results given in Table 4 show the average vertical height differences between the alveolar crest and the nasal cavity for the same tooth regions in panoramic views and CBCT sections.

The results of the analysis given in Table 5 suggest that the dental regions and interval lengths are not statistically independent from each other (*p* < 0.001). Specifically, for the central region, there is no statistical difference (*p* > 0.05) between the ranges [−4, −3], [−3, −2], [−2, −1], [−1, 0], and [0, 1], while the ranges [2, 3], [3, 4], and [4+] each have significantly different rates (*p* < 0.05). The ranges [2, 3], [3, 4], and [4+] have similar rates among themselves (*p* > 0.05). For the lateral region, the ratios of the ranges [−4, −3], [−3, −2], [−2, −1], [−1, 0], [0, 1], and [1, 2] were found to be similar to each other, while these rates were significantly different from the ranges [2, 3], [3, 4], and [4+]. Similar interpretations can be drawn for the canine and first premolar regions.

These statistical data lead us to the following conclusion. When panoramic radiographs and CBCT measurements made in the canine and first premolar regions are compared, the measurements made in panoramic radiographs give higher values than CBCT. In the central and lateral teeth regions, lower values are obtained in panoramic radiographs compared to CBCT. As can be understood from these data, different values can be obtained for the same region in 2D and 3D images.

## 4. Discussion

No study evaluating the incidence of nasal cavity perforations was found in the literature. It has been observed that virtual implants that are 12 and 14 mm in length are more likely to cause perforations in the nasal cavity in individuals with atrophic maxilla. When we looked at the case reports in the literature presenting complications due to nasal cavity perforations, we found that only one study provides information about the lengths of the implants that caused the complication [13]. The presented study draws attention to the possibility of a relationship between implant length and complications in the nasal cavity. On the other hand, some studies have proven that slightly perforating the nasal cavity to provide bicortical anchorage will increase primary stability [14,15,16,17,18]. In addition, it has been stated in the same studies that the thick nasal mucosa in this area will reduce the risk of complications [14,15,16,17,18]. In addition, sufficient experience and knowledge of the anatomy of the alveolar crest are required for bicortical implant placement in the anterior maxilla.

In developing countries, there is a trend to use high-precision three-dimensional imaging instead of two-dimensional imaging for stomatological diagnosis. However, due to cost and medical investment, local hospitals often do not have access to CBCT. Doctors have to perform implant surgery using panoramic radiographs to reduce risks. Therefore, it is clinically meaningful to know the possible differences between panoramic radiographs and CBCT images. Then, clinicians can estimate the actual bone measurement based on the panoramic radiograph measurement and possible differences to achieve ideal implant placement.

When the amount of impaction of implants placed in the anterior maxilla, which is another outcome of this study, was evaluated, an average value of 2.55 mm was found. This average was 3.17 mm for the canine region, which is significantly higher than other regions. A study by Pietrokovski et al., in which they analyzed the morphology of edentulous crests, showed that 47% of the crests with a knife edge that form in the residual alveolar crest were found in the anterior maxilla [19]. This result is consistent with this current study. In the knife-edge alveolar ridge, the coronal part of the implant must be placed slightly deeper than the ridge crest level in order to remain completely within the bone. This is an issue that should be taken into consideration in order to prevent perforations related to the nasal cavity.

**Figure 6:**
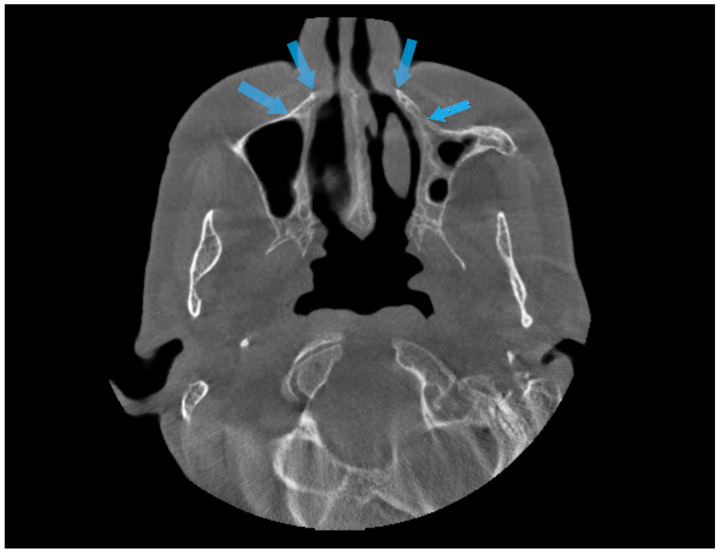
Blue arrows show cortical bone in the lateral wall of the nasal cavity and the medial wall of the maxillary sinus.

**Figure 7:**
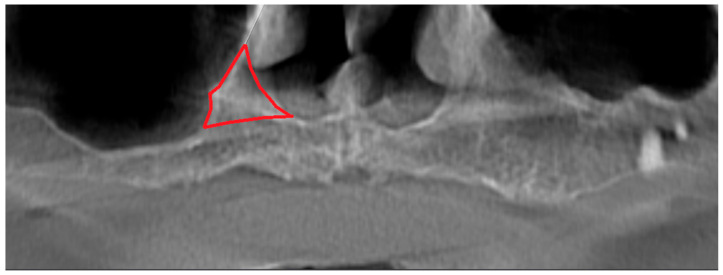
Red lines indicate the triangular region on the lateral side of the nasal cavity.

Panoramic radiographs may be insufficient to understand the anatomy of the nasal cavity. In their retrospective study, Park et al. evaluated patients who underwent sinus floor elevation with CBCT. As a result, they found that 13 of 72 patients accidentally perforated the nasal cavity [13]. In the same study, it was reported that two implants were lost, and the apical portions of the implants were surrounded by thin nasal mucosa with endoscopic visualization [13]. In this current study, significant differences were observed in vertical measurements made on panoramic and CBCT images. The measurements made on the panoramic radiograph for the canine and first premolar region were higher than the actual vertical height. Park et al. associated this condition with inferior meatus pneumatization in their study [13]. Yeom et al. attributed this situation to the following two factors: the nasal fossa in this region could not be detected because the horizontal radiopaque line of the hard palate was similar to or below the antrum floor, or because the cortical structure of the bone between the lateral wall of the nasal cavity and the medial wall of the maxillary sinus (Figure 6) was observed as a triangular shape, as if the bone volume was larger than the real three-dimensional volume [20]. The point we want to emphasize in our study is the triangular region on the lateral side of the nasal cavity (Figure 7). In individuals with atrophic maxilla, implants intended to be placed in the canine and first premolar tooth region are in the inferior part of this triangular area. They may cause various complications because the vertical amount of the residual alveolar crest is small, and this triangular region makes it difficult to fully diagnose the nasal cavity borders. For this reason, in cases of atrophic maxilla, even if sufficient bone is seen on panoramic radiography, applying CBCT will help prevent complications.

There are some limitations in this study. One of these was that the virtual implants were only placed at certain lengths and only measured a certain diameter with respect to a model of a single brand. In our study, nasal cavity perforations were associated only with vertical distance. However, the following has been overlooked: As the diameter of the implants increases, they may cause small perforations in the horizontal direction. In addition, the virtual implants placed in this study were 8, 10, 12, and 14 mm long. Different brands have implant options in intermediate sizes. Another limitation is that all CBCT and panoramic radiographs evaluated were obtained with a single brand of device. In different brands of devices, the transfer of bone density to the image and the sharpness of the demarcation lines may cause different measurements. This situation was also neglected in our study. Another issue was that the panoramic radiograph was obtained from CBCT sections. The reason for this is that if a second panoramic radiograph is taken from the patients, the environment where the X-ray is taken will be different, and a different technician will take the X-ray; with differences in positioning, we think that variables such as these will increase, and the differences in the measurements will increase depending on these variables.

## 5. Conclusions

Carrying out radiographic examination before implant surgery can prevent complications in individuals with atrophic maxilla. Implants with lengths of 12 and 14 mm may have the potential to create perforations in the anterior maxilla region. There are significant vertical length differences between panoramic and CBCT images of the same individuals. This difference is greater than the actual size in the panoramic radiograph in the canine and first premolar regions, while it is less than the actual size in the lateral and central incisor regions. Panoramic radiographs may be inadequate in understanding the three-dimensional anatomy of the maxilla and preventing nasal cavity perforations. Planning with virtual implants and fully understanding the anatomy of the nasal cavity on images obtained from CBCT can prevent complications.

## Figures and Tables

**Table 1:** Distribution of demographic attributes.

	Mean	Median	Standard Deviation
Age	60.81	60.00	11.39
		Count	Percentage
Gender	Male	28	47.5%
	Female	31	52.5%

**Table 2:** Comparison results of h3 lengths across different regions.

	Overall h3		F	*p*
	Mean	Standard Deviation		
Central	2.42 ^a^	1.58	9.880	<0.001
Lateral	2.36 ^a^	1.88		
Canine	3.17 ^b^	1.84		
First premolar	2.25 ^a^	1.42		

Different letters represent a significant difference for the corresponding region.

**Table 3:** Results of the investigation of the proportions of implant lengths and perforation conditions in the different tooth regions.

Implant Length and Region			Perforation Condition		Chi Square	*p*
			Perforation Exists	No Perforation		
8 mm	Region	Central	15 (25.9%)	103 (24.9%)	2.752	0.431
		Lateral	11 (19.0%)	107 (25.8%)		
		Canine	19 (32.8%)	99 (23.9%)		
		1st Premolar	13 (22.4%)	105 (25.4%)		
		Total	58	414		
10 mm	Region	Central	53 (29.9%)	65 (22.0%)	7.620	0.055
		Lateral	44 (24.9%)	74 (25.1%)		
		Canine	47 (26.6%)	71 (24.1%)		
		1st Premolar	**33 (18.6%)**	**85 (28.8%)**		
		Total	177	295		
12 mm	Region	Central	85 (27.6%)	33 (20.1%)	8.073	0.045
		Lateral	79 (25.6%)	39 (23.8%)		
		Canine	79 (25.6%)	39 (23.8%)		
		1st Premolar	**65 (21.1%)**	**53 (32.3%)**		
		Total	308	164		
14 mm	Region	Central	**105 (26.8%)**	**13 (16.3%)**	13.727	0.003
		Lateral	**105 (26.8%)**	**13 (16.3%)**		
		Canine	95 (24.2%)	23 (28.7%)		
		1st Premolar	**87 (22.2%)**	**31 (38.8%)**		
		Total	392	80		
Total	Region	Central	**258 (27.6%)**	**214 (22.5%)**	16.381	<0.001
		Lateral	239 (25.6%)	233 (24.4%)		
		Canine	240 (25.7%)	232 (24.3%)		
		1st Premolar	**198 (21.2%)**	**274 (28.8%)**		
		Total	935	953		

Bold indicates statistical significance.

**Table 4:** Descriptive statistics of h1–h2 difference for each region.

	Central	Lateral	Canine	1st Premolar
Mean	−0.2021	0.0778	3.5331	3.7251
Minimum	−3.15	−3.00	0.80	0.40
Maximum	2.30	1.90	7.00	7.65

**Table 5:** Results of comparisons of different ranges within the same dental region.

		Range											
		−4 < h_dif_ < −3	−3 < h_dif_ < −2	−2 < h_dif_ < −1	−1 < h_dif_ < −0	0 < h_dif_ < 1	1 < h_dif_ < 2	2 < h_dif_ < 3	3 < h_dif_ < 4	4 < h_dif_	X^2^	*p*	cc
Region	Central	3 ^a^	5 ^a^	5 ^a,b^	21 ^a^	17 ^a^	5 ^a,b^	2 ^a,b,c^	1 ^b,c^	0 ^c^	201.627	<0.001	0.679
	Lateral	2 ^a,b^	3 ^a,b,c^	9 ^b^	14 ^a,b^	18 ^a,b^	11 ^a,b^	1 ^a,b,c^	0 ^c,d^	1 ^d^			
	Canine	0 ^a,b,c^	0 ^a,b,c^	0 ^b,c^	0 ^c^	1 ^c^	4 ^a,b,c^	8 ^a^	13 ^a^	33 ^a,b^			
	1st Premolar	0 ^a,b,c^	0 ^a,b,c^	0 ^b,c^	0 ^c^	1 ^c^	5 ^a,b,c^	4 ^a,b,c^	10 ^a,b^	39 ^a^			
	Total	5	8	14	35	37	25	15	24	73			

Note: h_dif_ represents h1–h2. Different letters represent a significant difference for the corresponding region.

## Data Availability

The datasets used and/or analyzed in this current study are available from the corresponding author upon reasonable request.
